# Applicability of Deep Learning to Dynamically Identify the Different Organs of the Pelvic Floor in the Midsagittal Plane

**DOI:** 10.1007/s00192-024-05841-0

**Published:** 2024-06-24

**Authors:** José Antonio García-Mejido, David Solis-Martín, Marina Martín-Morán, Cristina Fernández-Conde, Fernando Fernández-Palacín, José Antonio Sainz-Bueno

**Affiliations:** 1 Department of Obstetrics and Gynecology, Valme University Hospital, Seville, Spain; 2 Department of Surgery, Faculty of Medicine, University of Seville, Seville, Spain; 3 Department of Computer Science and Artificial Intelligence, Faculty of Mathematics, University of Seville, Seville, Spain; 4 Department of Statistics and Operational Research, University of Cadiz, Cadiz, Spain

**Keywords:** Deep learning, Pelvic floor, Ultrasonography, Convolutional neural network, Artificial intelligence, Levator ani muscle

## Abstract

**Introduction and Hypothesis:**

The objective was to create and validate the usefulness of a convolutional neural network (CNN) for identifying different organs of the pelvic floor in the midsagittal plane via dynamic ultrasound.

**Methods:**

This observational and prospective study included 110 patients. Transperineal ultrasound scans were performed by an expert sonographer of the pelvic floor. A video of each patient was made that captured the midsagittal plane of the pelvic floor at rest and the change in the pelvic structures during the Valsalva maneuver. After saving the captured videos, we manually labeled the different organs in each video. Three different architectures were tested—UNet, FPN, and LinkNet—to determine which CNN model best recognized anatomical structures. The best model was trained with the 86 cases for the number of epochs determined by the stop criterion via cross-validation. The Dice Similarity Index (DSI) was used for CNN validation.

**Results:**

Eighty-six patients were included to train the CNN and 24 to test the CNN. After applying the trained CNN to the 24 test videos, we did not observe any failed segmentation. In fact, we obtained a DSI of 0.79 (95% CI: 0.73 – 0.82) as the median of the 24 test videos. When we studied the organs independently, we observed differences in the DSI of each organ. The poorest DSIs were obtained in the bladder (0.71 [95% CI: 0.70 – 0.73]) and uterus (0.70 [95% CI: 0.68 – 0.74]), whereas the highest DSIs were obtained in the anus (0.81 [95% CI: 0.80 – 0.86]) and levator ani muscle (0.83 [95% CI: 0.82 – 0.83]).

**Conclusions:**

Our results show that it is possible to apply deep learning using a trained CNN to identify different pelvic floor organs in the midsagittal plane via dynamic ultrasound.

**Supplementary Information:**

The online version contains supplementary material available at 10.1007/s00192-024-05841-0.

## Introduction

Transperineal ultrasound represents an important advance in the study of pelvic floor pathology. The imaging methodology used in this type of diagnostic test is one of its characterizing qualities because it is clearly standardized in the literature [1]. To diagnose multiple pelvic floor dysfunctions, the midsagittal plane is used as a reference for the study of the pelvic floor with 2D transperineal ultrasound. This plane allows the analysis of the pubic symphysis, urethra, urinary bladder, vagina, uterus, anal canal, rectum, and levator ani muscle in the same ultrasound section [1]. In addition, it allows us to dynamically study the different organs and associated dysfunctions, such as pelvic organ prolapse. However, this technique involves manual measurements, which require the time and experience of the sonographer who performs them, leading to significant variations in qualification between observers [2]. Therefore, a technology that allows dynamic analysis of different pelvic organs from the midsagittal plane will accelerate the use of this technique, making it more applicable in clinical consultations.

In contrast to the classic and manual methods of ultrasound of the pelvic floor, some studies have used artificial intelligence (AI) to identify different pelvic floor structures [3–6]. Interest in applying AI to the field of gynecology is increasing [7], leading to a change in the vision of medicine and posing new challenges [7]. AI refers to the ability of a computer program to perform reasoning processes similar to human intelligence [8]. Deep learning (DL) is an AI subcategory in which algorithms are used to enable computers to learn on their own and perform tasks similar to those of human beings. In the case of the pelvic floor, DL has been used in the axial plane of minimum dimensions to study the pelvic floor musculature [3–6]. These studies are based on very specific static images that allow analysis of the levator ani muscle and measurements of the hiatus only [3–6]. However, analyzing static images of the pelvic floor may be insufficient as there are dysfunctions, such as pelvic organ prolapse, that require dynamic study to obtain an ultrasound diagnosis. In addition, the dynamic assessment of pelvic floor organs allows us to analyze the multicompartmental relationships between different pelvic organs during Valsalva maneuvers. Therefore, we propose that convolutional neural networks (CNNs) could be very useful in facilitating dynamic and multicompartment study of the pelvic floor in the midsagittal plane. Based on this premise, we posed the following question: can a CNN identify the different organs of the different compartments of the pelvic floor through dynamic ultrasound study of the midsagittal plane?

The objective of our work was to create and validate the usefulness of a CNN to identify the different organs of the pelvic floor in the midsagittal plane through dynamic ultrasound study and to establish its concordance with an expert observer.

## Materials and Methods

An observational and prospective study was carried out with 110 patients. The patients were recruited consecutively in general gynecology consultations from 1 April 2023, to 31 July 2023. Patients did not need to suffer from pelvic floor pathologies to qualify. However, patients who underwent surgery or had a history of pelvic floor dysfunction and patients with problems that made it difficult to perform a correct Valsalva maneuver were excluded. All patients were gynecologically evaluated before being included in the study to rule out pelvic floor dysfunction. The clinical parameters studied were age, weight, height, body mass index (BMI), parity, menopausal status, and age at menopause.

### Ultrasound Examination and Segmentation

The transperineal ultrasound scans were performed by an expert pelvic floor sonographer on a Canon i700 Aplio® (Canon Medical Systems, Tokyo, Japan) with a PVT-675 MV 3D abdominal probe. The images were acquired with patients in the dorsal lithotomy position with their hips flexed and following the guidelines previously established in the literature [1]. The probe (covered with a protective sleeve) was carefully placed in the perineum, less than 1 cm from the pubic symphysis, with both labia minora on the sides of the transducer. The midsagittal plane included a view of the symphysis pubis, urethra, bladder, vagina, uterus, anus, rectum, and levator ani muscle. To obtain a good image of the uterine fundus, low frequencies were used to capture a complete image of all the organs. The orientation of the ultrasound videos was established such that the cranioventral region was on the left and the dorsocaudal region was on the right (Video 1) [9]. Before the video was captured and stored, the patient was trained to perform the Valsalva maneuver correctly. A video was made of each patient that included the midsagittal plane of the pelvic floor at rest and the change in the pelvic structures during the Valsalva maneuver. After saving the captured videos, we manually labeled the different organs in each video (Video 1). Labeling was performed by correcting the movement of the different organs during the Valsalva maneuver (Video 1). Tagging was performed by two independent scanners and supervised by an expert scanner (JAGM).

Data were labeled using the free software CVAT, developed by Intel specifically for annotating both images and videos. CVAT offers a variety of annotation shapes and types, including labels, bounding boxes, polygons, polylines, dots, and cuboids.

For each video, the sonographer selected a series of frames based on observable changes in the image resulting from patient maneuvers. These frames were annotated by outlining polygons around each organ of interest. As only a subset of frames was annotated, labels for the remaining frames needed to be provided. In this study, linear interpolation between two adjacent annotated frames was used to generate labels for the remaining frames.

A total of 110 videos were tagged and randomized into two groups. The first group comprising 86 tagged videos served as the CNN training set, whereas the second group of 24 videos was used for CNN validation.

### Algorithm

Prior to training the networks, the ultrasound images were preprocessed to eliminate the background, converted to grayscale and resized to 128 × 128 pixels. The data were input into the network in their raw format. No preprocessing was applied to the images to remove noise or to adapt to the variability of the distributions. Instead, these tasks were delegated to the neural network.

In total, 15,932 raw images (before applying data augmentation preprocessing) were distributed across the training, validation, and test sets. Data splitting was performed on a per-patient basis to mitigate the risk of overfitting. This approach ensures that the model generalizes well to unseen patient data, thereby enhancing the reliability of the results.

**Fig. 1:**
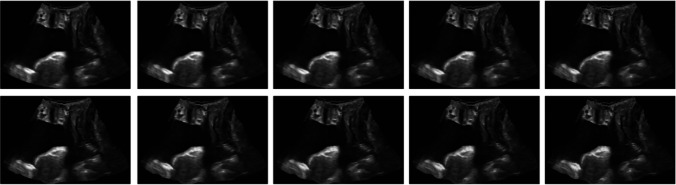
An example of the elastic transformations applied to a video frame

Various data expansion techniques, including rotation, scaling, translation, and elastic transformations, were tested. The rotation range applied was between −180 and 180°. The scaling factor ranged between 0.8 and 1.5, and the translation sliding was 10% from the center of the image in all directions. These ranges were applied randomly. Figure 1 shows an example of the elastic transformations applied to a video frame.

**Fig. 2:**
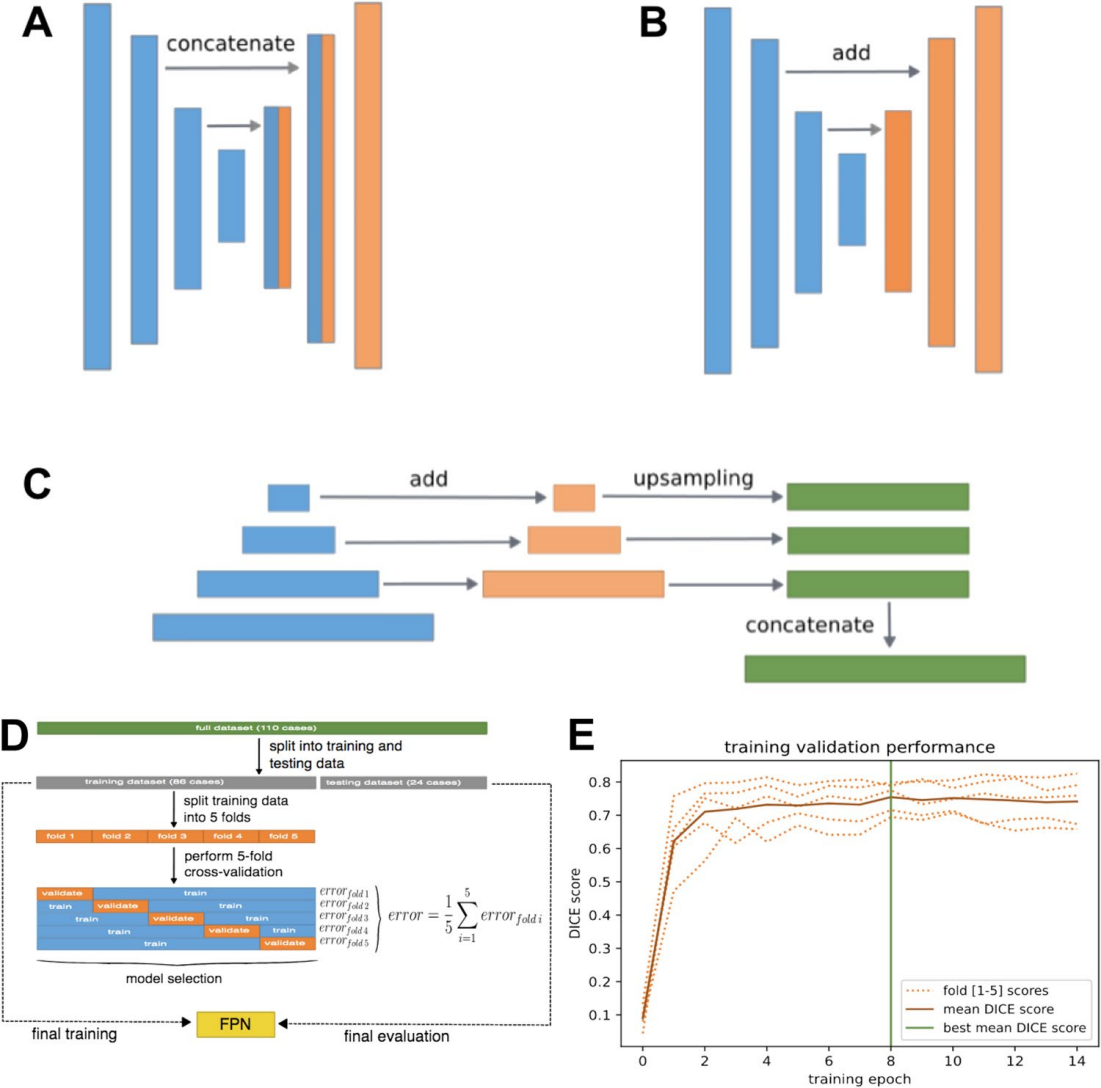
Three different architectures were used. **A** UNet architecture with skip connections that use concatenation. **B** LinkNet, in which the sum operation is used in skip connections. **C** FPN shows the validation for the selection of the best network. **D** Data partitioning, cross-validation, and final training methodology. **E** Model performance during training on the validation sets of each fold. The *green vertical line* indicates the best average performance obtained

In this work, three different architectures were tested: UNet, FPN, and LinkNet (Fig. 2). These architectures are well-known models utilized for image segmentation tasks. They were selected to evaluate their performance on 2D ultrasound images in the midsagittal plane, with the aim of estimating the regions of eight different organs. UNet [10] was designed expressly for segmenting medical images and has been used extensively in this context. It comprises two parts: an encoder and a decoder. The encoder performs a dimensionality reduction in which it extracts the useful feature information that will be used by the decoder. In this phase, as the network deepens, the spatial information is reduced. The decoder performs a dimensional expansion in which characteristic information is combined with spatial information to construct the output. These connections between the encoder and decoder are known as skip connections. LinkNet [11] has a very similar structure to UNet, except that a sum operation is applied in the connections between the encoder and decoder. This difference, such as the use of separable convolutions and 1 × 1 convolutions, achieves better computational efficiency and training speed and maintains good performance in segmentation precision. Finally, the feature pyramid network (FPN) [12] reuses the feature maps of each stage of the decoder to concatenate them after unification of the dimensions and to obtain the final segmentation.

For each architecture, 11 different backbones were tested (ResNet50, VGG16, VGG19, DenseNet121, BeingsNet50, ResNeXt50, BeingsNeXt50, Inceptionv3, InceptionResNetv2, EfficientNetv5, and EfficientNetv7). The backbone is a network that is used as an encoder and that can be pretrained. In this work, the weights of the backbones were randomly initialized.

To avoid possible overfitting when selecting the best network, cross-validation was applied (Fig. 2). Five folds were used; thus, the model was trained on 69 cases and validated on 17 cases. As a criterion for stopping the training, early stopping was used with a stopping criterion of five times without improvement in the mean of the validation errors of the five folds. In total, 165 networks were trained (3 networks × 11 backbones × 5 transformations).

The networks were trained with a cost function (loss function) that combines the focal function with the Dice function. The Dice function, which measures the level of overlap of the prediction and the labeled region, assigns a weight of 1 to the region of each recognized organ and a weight of 0.5 to the background. The focal cost function [13] is an improvement of the cross-entropy function to address unbalanced data and focuses more on samples that are difficult to classify.

**Fig. 3:**
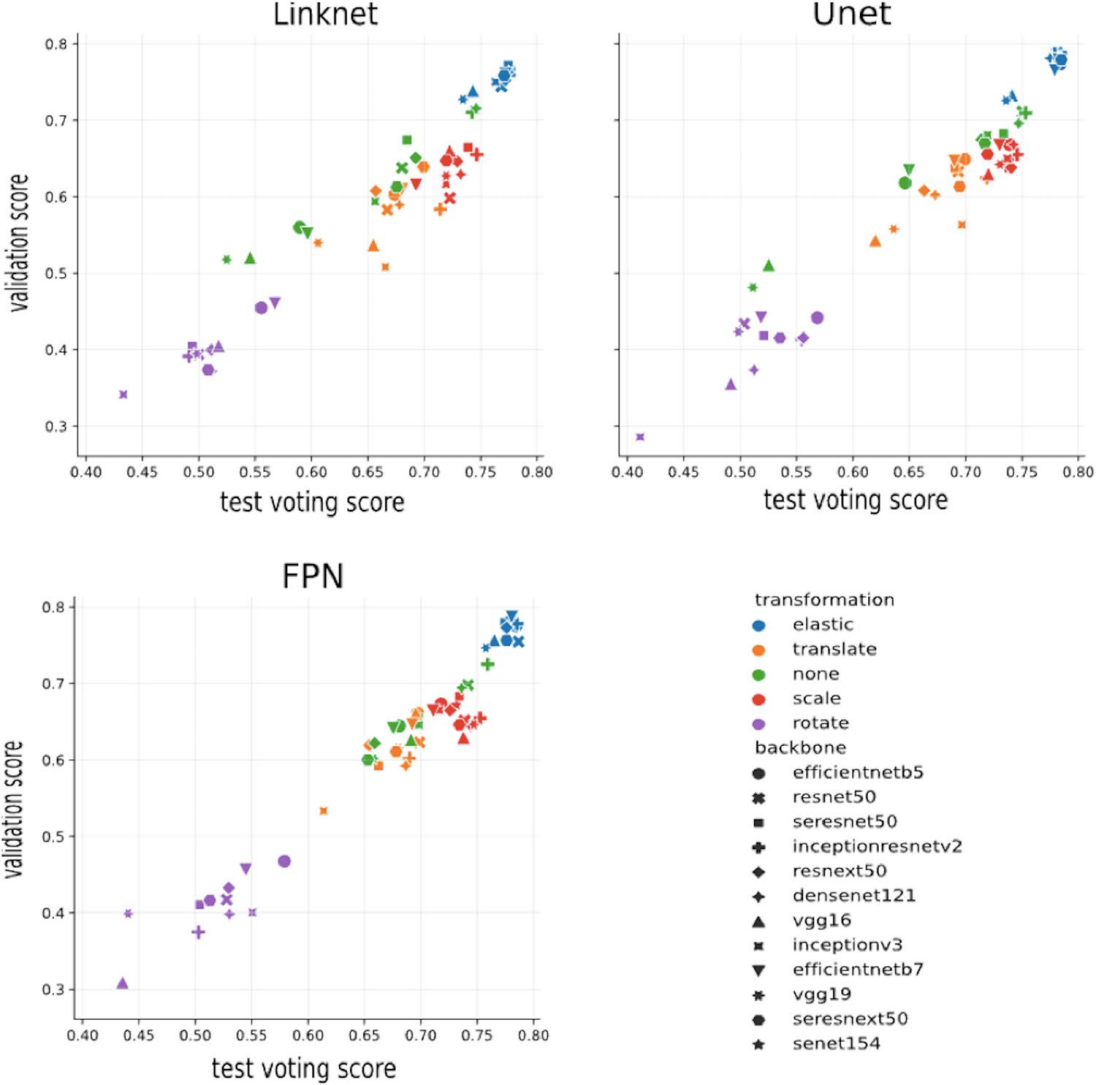
The graph compares the validation and test Dice Similarity Index scores obtained for each pair of network architecture and backbone with a specific data augmentation

Figure 3 displays the results for each architecture compared with each backbone and the data augmentation applied. The best model was found to be FPN + ResNet50, with elastic transformations at a probability of 50% (the elastic transformation was applied to 50% of the training samples). This model was trained with 86 cases for the number of epochs determined by the stop criterion via cross-validation.

The model was trained to independently estimate the region of each organ in every frame. Once trained, the model was applied to predict all frames within the test videos, and the scores are computed by measuring the mean performance per video.

All networks were trained using an NVIDIA GTX 1080Ti GPU installed on an Intel Core i5-7500 3.40 GHz CPU running Ubuntu 20 with 32 GB of RAM. To implement the networks, the Keras framework and the segmentation models package were used [14].

### CNN Evaluation Metrics

The Dice Similarity Index (DSI) was used for CNN validation. The DSI determines the similarity between manual labeling and the CNN; a DSI = 0 indicates no overlap between the manual segmentation and the CNN, whereas a DSI = 1 indicates maximum overlap between the segmentations. The DSI is calculated using the following formula: (2 | X ∩ Y |) ÷ (| X | +| Y |), where X and Y are the two segmentations. In addition, the intersection over union (IoU) was used, which describes the level of overlap between two boxes, that is, the prediction box and the real bounding box. The greater the overlap, the greater the IoU. The IoU is calculated by determining the Jaccard index: IoU = (ANB) ÷ (AUB) or (I) ÷ (U).

### Agreement with the Expert Observer

**Fig. 4:**
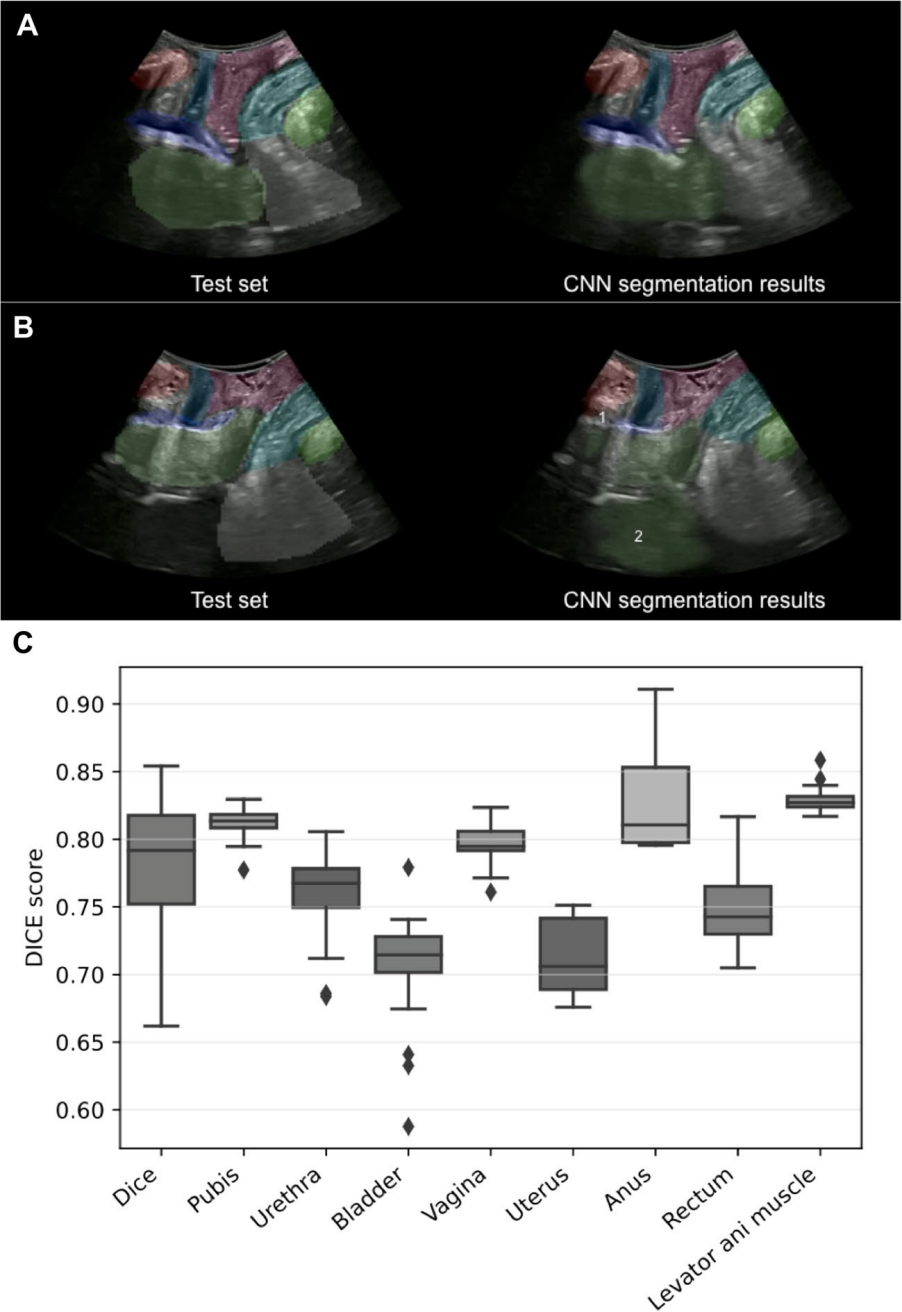
The image shows correct recognition (**A**) and defective recognition (**B**) of the different organs of the pelvic floor in the midsagittal plane by the convolutional neural network according to the expert explorer. In **B** there is a partial lack of recognition of the urinary bladder owing to the pubic shadow (*1*) and poor delimitation of the posterior uterine surface owing to the similar echogenicity that the uterus can present with the intestinal loops (*2*). Box plot showing the general Dice Similarity Index (DSI; *DICE*) and the DSI of the different organs (**C**)

An expert observer (JAGM) compared the manually tagged videos with the CNN segmentations of the 24 videos used for validation. The expert observer considered the image to be correctly recognized by the CNN when its segmentation completely identified the organ under study; that is, the segmented image was similar to the image that was manually labeled (Video 1). A CNN segmentation that partially identified the organ (Fig. 4) or a segmentation image that was different from the image that was manually labeled (Fig. 4) was classified as incorrect.

### Statistical Study

Statistical analysis was performed using the IBM SPSS Statistics version 26 program (IBM, Armonk, NY, USA). The data were reviewed before statistical analysis. To describe the numerical variables, means and standard deviations (SDs) were used in the case of an asymmetric distribution. For example, the medians and percentiles (p25 and p75) were used, and the qualitative variables were expressed as percentages.

### Ethical Approval

The study was conducted in accordance with the Declaration of Helsinki (as revised in 2013). The study (0625-N-23) was approved by the local Ethics and Research Committees in April 2023. All patients provided written informed consent before starting the study.

## Results

The general characteristics of the patients whose videos were included for training (*n* = 86) and for testing the CNN (*n* = 24) are listed in Table 1.

**Table 1:** The general characteristics of the patients included to train (*n* = 86) and to test the convolutional neural network (*CNN*) (*n* = 24)

	Patients included to train the CNN (*n* = 86)	Patients included to test the CNN (*n* = 24)	*p*	95% CI
Age, mean (± SD)	44.7 ± 12.2	43.4 ± 15.5	0.718	−8.3; 5.7
Weight, mean (± SD)	69.9 ± 17.5	67.9 ± 18.2	0.659	−13.0; 9.0
Height, mean (± SD)	161.7 ± 5.7	161.8 ± 7.4	0.957	−4.0; 4.2
BMI, mean (± SD)	26.9 ± 6.3	25.6 ± 5.9	0.515	−5.4; 2.7
Parity, mean (± SD)	1.5 ± 1.2	1.4 ± 1.1	0.969	−1.0; 1.0
Menopause, *n* (%)	13/63 (20.6%)	4/15 (26.7%)	0.729	−31.7%; 16.0%
Menopause age, mean (± SD)	52.0 ± 2.4	51.8 ± 2.8	0.883	−3.0%; 2.6%

After applying the trained CNN to the 24 test videos, we did not observe any failed segmentation. In fact, we obtained an IoU of 0.66 (95% CI: 0.60 – 0.70) and a DSI of 0.79 (95% CI: 0.73 – 0.82) as the median of the 24 test videos. When we studied the organs independently, we observed differences in the DSI of each organ. The poorest DSIs were obtained for the bladder (0.71 (95% CI: 0.70 – 0.73)) and the uterus (0.70 (95% CI: 0.68 – 0.74)), whereas the highest DSIs were obtained for the anus (0.81 (95% CI: 0.80 – 0.86)) and the levator ani muscle (0.83 (0.82; 0.83); Fig. 4; Table 2). The interpretation made by the expert sonographer regarding the performance of the trained CNN on the 24 test videos is shown in Table 3. The expert sonographer determined that the trained CNN recognized all the organs except the bladder and uterus at rest (including the first second of the video where the musculature did not move, which correctly identified it in 83.3% and 75% respectively), during the Valsalva (including the second of the video when the levator ani muscle approached the pubis, which correctly identified it in 91.7% and 79.2% respectively), and throughout the video (including good recognition of the different organs during all the seconds of the video, which identified it correctly in 83.3% and 75% respectively; Fig. 4; Video 1).

**Table 2:** The values of the intersection over union (*IoU*), general Dice Similarity Index (*DSI*; *DICE*), and the DSI of the different organs are shown

	Median (P_25_; P_75_)	95% CI median
IoU	0.66 (0.60; 0.70)	0.55–0.75
General DSI (DICE)	0.79 (0.73; 0.82)	0.66–0.85
Pubis DSI	0.81(0.80; 0.82)	0.78–0.83
Urethra DSI	0.77(0.75; 0.78)	0.68–0.81
Bladder DSI	0.71(0.70; 0.73)	0.59–0.78
Vagina DSI	0.79(0.79; 0.81)	0.76–0.82
Uterus DSI	0.70(0.68; 0.74)	0.68–0.75
Anus DSI	0.81(0.80; 0.86)	0.79–0.91
Rectum DSI	0.74(0.72; 0.77)	0.70–0.82
Levator ani muscle DSI	0.83(0.82; 0.83)	0.82–0.86

**Table 3:** Interpretation made by the expert explorer of the accuracy of the trained convolutional neural network (*CNN*) on the 24 test videos at rest, Valsalva, and throughout the video

	Correct recognition of CNN at rest		Correct recognition of CNN in Valsalva		Correct recognition of CNN throughout the video	
	Percentage	95% CI	Percentage	95% CI	Percentage	95% CI
Pubis	100 (24/24)	–	100 (24/24)	–	100 (24/24)	–
Urethra	100 (24/24)	–	100 (24/24)	–	100 (24/24)	–
Bladder	83.3 (20/24)	67.3–99.4	91.7 (22/24)	79.7–100.0	83.3 (20/24)	67.3–99.4
Vagina	100 (24/24)	–	100 (24/24)	–	100 (24/24)	–
Uterus	75 (18/24)	56.3–93.7	79.2 (19/24)	61.6–96.7	75 (18/24)	56.3–93.7
Anus	100 (24/24)	–	100 (24/24)	–	100 (24/24)	–
Rectum	100 (24/24)	–	100 (24/24)	–	100 (24/24)	–
Levator ani muscle	100 (24/24)	–	100 (24/24)	–	100 (24/24)	–

## Discussion

In this work, we designed a CNN that allows us to recognize all pelvic organs in the midsagittal plane via transperineal ultrasound (Video 2). We obtained a CNN that presented a median for the general DSI of 0.79 (0.73; 0.82). However, this DSI varies depending on the organ studied, obtaining the best results with a median DSI above 0.8 in the organs that are closest to the transducer (pubis, vagina, anus, and levator ani muscle) and the worst in the organs that are more distant, such as the urinary bladder and the uterus. The expert sonographer determined the results in accordance with those observed in the DSI, noting that the organs most severely delimited by the CNN were the bladder and the uterus. This worsening of the delimitation was due mainly to the acoustic shadow produced by the pubis and to the similarities in the echogenicity that the uterus can present with the intestinal loops, as shown in Fig. 4B. Another aspect that may influence the DSI of the urinary bladder is that we do not propose standard bladder filling during the examination. We aimed to determine whether the CNN could recognize the urinary bladder in the different filling situations in which it can be found. This aspect makes DSI in the urinary bladder worse when it is completely empty (Fig. 4B) and improves when there is a minimum amount of urine.

To our knowledge, this study is the first to use a CNN to identify pelvic floor organs in the midsagittal plane via transperineal ultrasound. Therefore, we cannot compare our results with those of other authors. Previous authors have applied DL to view other structures of the pelvic floor on ultrasound, with disparate ISD results [4–6, 15]. Studies that examined the hiatus area of the elevator using DL reported DSIs greater than 0.9 [4, 5, 15, 16], establishing a value of 0.94 in the case of the urogenital hiatus [17]. However, when using the DL to study solid structures, as in the case of the levator ani muscle, the DSI is lower, ranging between 0.6 and 0.77 [4, 18, 19]. The reason for this may be that solid structures present less contrast with neighboring structures and are more difficult to delimit. These results are consistent with those obtained in our work. However, unlike previous studies in which only the levator ani muscle was studied in static images [4, 6], we studied eight organs simultaneously and dynamically.

The application of AI to the field of gynecology has shown usefulness for assessing breast lesions [20, 21], evaluating adnexal masses [22], determining the probability of metastasis in endometrial cancer [23], and evaluating urodynamic stress incontinence with ultrasound [3]. Our work opens up an important field of study as our CNN can influence the training of future sonographers, helping them to learn about the ultrasound capture process of the midsagittal plane, a process that, until recently, has required an expert sonographer [24]. Additionally, by demonstrating that a CNN can dynamically detect the different components that we observe via 2D transperineal ultrasound of the pelvic floor, we will be able to study different pathologies, such as pelvic organ prolapse. Until recently, ultrasound was used to diagnose significant prolapse in each compartment in relation to the postero-inferior margin of the symphysis pubis during the Valsalva maneuver, and a differential diagnosis was made within each compartment [25, 26]. However, these diagnoses depend on different measurements [25], software diagnostics [27, 28], or knowledge about the aspect of the morphology that the organs present during the Valsalva maneuver [29]. These aspects require the time and experience of the sonographer who performs the ultrasound and can be difficult to provide in a typical consultation. The use of DL to identify pelvic organ prolapse has been previously suggested in the literature [30]. Furthermore, DL has shown that it is possible to simultaneously diagnose three types of prolapse based on pelvic floor stress MRI [31]. However, the latter study was based on static images, unlike the dynamic model proposed in this work. Therefore, based on our results, we propose that the use of DL in this field will be useful. For example, we intend to apply the technology shared in this study to patients with pelvic organ prolapse. Now that we have shown that a CNN can reliably detect different organs dynamically in healthy women, the next step will be to analyze women with pelvic floor dysfunctions and retrain the CNN, an aspect on which we are currently working.

The main strength of our study lies in establishing the basis for dynamically identifying all pelvic floor organs in the midsagittal plane with a CNN. In addition, we present results similar to those previously reported when applying DL to determine the muscular structures of the pelvic floor [4, 6]. However, to our knowledge, our study is the first to use a CNN dynamically and not with an isolated image. This innovation could represent an advance for the typical clinic, as the incorporation of this technology into ultrasound equipment would guarantee capture of the midsagittal plane directly, helping the sonographer to obtain correct study of the reference plane. However, the main challenge concerning implementation relates to the manufacturers of ultrasound equipment as they would be responsible for launching this initiative. Another positive contribution of this study is that the manual tagging of our videos was carried out by different sonographers and supervised by an expert sonographer, thus avoiding possible biases. The main weakness that we observed in the CNN is that there are differences in the DSI between the different organs owing to the echogenicity and positioning that the organs present during a dynamic study. We believe that this aspect can be improved by training our CNN with a greater number of cases. Another weakness of our work is the inability to determine whether the CNN can identify different organs depending on the clinical characteristics of the patient. We have not been able to establish whether recognition varies depending on intrinsic factors of the patients that a priori influence the echogenicity of the tissues, such as BMI, age, tissue hydration, or pelvic floor dysfunction. In addition, we used 110 videos (15,932 raw images) to train and validate this CNN; to generalize our results, we would need a larger population. This possibility suggests future research directions to create a CNN that can respond to these aspects.

## Conclusions

We have shown that it is possible to apply DL using a trained CNN to identify the different pelvic floor organs in the midsagittal plane via dynamic ultrasound.

## Supplementary Information

Below is the link to the electronic supplementary material.

Supplementary file1 (MP4 12164 KB)

Supplementary file2 (MP4 3625 KB)
